# How Sustainable Are Hospital Menus in the United Kingdom? Identifying Untapped Potential Based on a Novel Scoring System for Plant‐Based Provisions

**DOI:** 10.1111/jhn.70019

**Published:** 2025-02-03

**Authors:** Isabelle Sadler, Alexander Bauer, Shireen Kassam

**Affiliations:** ^1^ Plant‐Based Health Professionals UK London UK; ^2^ Institute for Prevention and Cancer Epidemiology, Faculty of Medicine and Medical Center University of Freiburg, Freiburg im Breisgau Germany; ^3^ King's College London University of Winchester Hampshire UK

**Keywords:** environmental sustainability, hospital menus, net‐zero healthcare, plant‐forward menus

## Abstract

**Background:**

Adopting plant‐forward diets is essential for achieving climate targets. As the second‐largest provider of public sector meals in the UK, the National Health Service (NHS) can significantly reduce its environmental impact by transitioning to plant‐forward menus, contributing to its goal of being a net‐zero healthcare service by 2045. This study evaluates the extent to which NHS hospitals currently align with sustainable practices by assessing the plant‐forward nature of in‐patient menus.

**Methods:**

Green Plans from 40 hospital trusts were analysed to assess commitment to plant‐forward, lower‐emission menus. Freedom of Information requests were sent to 50 NHS trusts, and 36 menus from the spring/summer season of 2024 were analysed. A novel scoring system was developed to assess the hospital menus, with subscores reflecting the availability of plant‐based meals, ruminant‐meat meals, and menu strategies to encourage plant‐forward choices.

**Results:**

Green Plans showed limited commitment to increasing plant‐based food options. Hospital menus scored poorly overall (average score of 20/100, range: 9–38). The lowest subscores were observed in the provision of fully plant‐based meals and nudging techniques. The provision of ruminant meat varied (subscore range: 0–100) and all hospitals included processed meat on their menu. Hospitals with outsourced catering scored higher than those with in‐house catering.

**Conclusion:**

Despite national recommendations to shift towards plant‐forward diets, NHS hospitals currently show little commitment and provide limited offerings in this regard. The novel scoring system offers a practical framework for monitoring progress and guiding hospitals towards environmentally sustainable, plant‐forward menus.

## Summary

- NHS hospitals have underutilised potential to reduce their environmental impact by shifting towards plant‐forward menus, contributing to climate targets and sustainability goals.
- The novel scoring system developed in this study provides an effective tool to assess and monitor the sustainability of menus in healthcare, focusing on plant‐based provisions.
- While NHS hospitals currently show limited commitment to plant‐forward diets, strategic improvements in menu design, the introduction of plant‐forward options, and nudging techniques could drive change towards more sustainable meal offerings.

## 1 Introduction

The 2024 Lancet Countdown on health and climate change reported that we are facing unprecedented threats to health and survival from the rapidly changing climate and delayed action to address it [1]. Dietary shifts are crucial in mitigating these threats, as global food systems contribute to one‐third of all anthropogenic greenhouse gas emissions (GHGEs) [2]. Red meat and dairy alone accounted for 56% of agricultural emissions in 2021 [1], while in the UK excessive consumption of these foods was associated with 38,500 deaths [1]. Additionally, processed meat is classified as a Group 1 carcinogen and is associated with an increased risk of all‐cause mortality [3, 4]. In Europe, transitioning to plant‐forward diets could reduce food‐associated GHGEs by up to 50%, cut land use by up to 62% [5], and reduce the risk of all‐cause mortality by up to 63% and cancer risk by up to 39% [5].

Global recognition of the need for health services to reduce their carbon footprint is growing. In 2021, the Global Road Map for Health Care Decarbonization identified seven priority actions for the healthcare sector to achieve net zero emissions [6]. The fourth was to provide healthy and sustainable food from climate‐resilient agriculture, which includes a reduced reliance on meat and dairy and the implementation of plant‐forward menus. This is echoed by the 2024 climate and health policy priorities for the UK developed by the UK Health Alliance on Climate Change [7]. Of the three critical priorities, investing and funding rapid decarbonisation and climate resilience in the UK National Health Service (NHS) is the first, which includes the adoption of a predominantly plant‐based dietary approach [7]. These recommendations align with the NHS' commitment to preventative healthcare as outlined in its Long‐Term Plan [8], and in achieving its goal of net zero carbon emissions by 2045 [9]. In England, NHS trusts are required to develop their own Green Plans, which outline their personal commitments and actions to support the NHS in becoming a net‐zero healthcare system [10].

The NHS is the second‐largest provider of meals in the UK public sector, serving 140 million inpatient meals annually [11]. NHS food and catering services are estimated to produce 1543 kt CO2e each year, accounting for approximately 6% of total NHS emissions [9], making these services a significant area for climate action. In addition to meeting the nutritional requirements of patients and preventing malnutrition, hospital menus must now also consider sustainability to meet NHS' net zero targets. Existing national guidelines, including the British Dietetic Association (BDA) Nutrition and Hydration Digest [12], the BDA One Blue Dot [13] and the NHS England National Standards for Healthcare Food and Drink [14] recognise the importance of sustainability. These guidelines acknowledge the lower environmental impact of plant‐based meals [12, 13], make recommendations to reduce animal‐based meat content, and implement ‘nudging’ techniques to encourage patients to choose plant‐based options [12]. However, these are voluntary recommendations, and the extent to which they are implemented across NHS hospitals remains unclear.

Nudging techniques alter the decision‐making context to change behaviour and have proven effective in changing food choices towards healthier or lower‐emission options [15, 16, 17]. In their playbook, The World Resources Institute (WRI) outlines 90 techniques for promoting plant‐based options, such as increasing the ratio of plant‐rich to meat‐based dishes on the menu, making all side dishes and extras on the menu plant‐rich only, and removing unappealing language describing plant‐rich dishes from menus [18].

Despite the recognised benefits of plant‐forward diets and guidance to increase their availability and consumption, adherence and implementation of these recommendations by NHS hospitals is unclear. To our knowledge, no studies have examined the provision of plant‐based food or red and processed meat in NHS hospitals or their compliance with the recommendations in the NHS Food Review and the BDA Nutrition and Hydration Digest. This study introduces a novel scoring system to evaluate current inpatient hospital food options, focusing on (A) plant‐based versus animal‐based offerings, and (B) the use of nudging techniques to encourage lower‐emission choices. The scoring system is designed to rank each hospital menu both overall and with respect to individual measures and thus allows for the identification of underutilised potential for further improving sustainability.

## 2 Materials and Methods

### 2.1 Study Design

The aim of this study was to assess the extent to which inpatient hospital menus in the UK have adopted environmental sustainability recommendations to promote a plant‐forward food environment. We analysed the standard inpatient menus based on contacting a representative selection of NHS hospitals across various regions of the UK, from hospitals with both in‐house and contracted out catering services.

The analysis focused on main meals served at lunch and dinner, and smaller meals such as sandwiches, soups, jacket potatoes, salads, and lighter appetite options. Starters and desserts were excluded due to their limited variety and generally being vegetarian in most menus. In cases where smaller meal items constituted the evening meal, these were recorded as the evening meal offerings.

The focus in this study was on the default menu provided, which should usually be appropriate for most hospital patients. While many hospitals offer specialised menus for specific dietary needs – often including vegan diets – consistently comparing these diverse specialised menus is challenging. No structured data was available on how proactively patients must request these menus in different hospitals and how often such specialised menus were handed out, though it is generally understood in NHS settings that patients actively request specialised menus. Since the default menu has by far the most impact and best reflects the overall effort of each hospital to reduce food‐based carbon emissions, we only scored these menus.

### 2.2 Sampling

A list of all NHS Trusts was obtained from the NHS website, split by region and Integrated Care Systems (ICS) [19]. The list of Trusts in each ICS was refined to include all teaching and general hospitals, as this is where most inpatient meals are served. From this refined list, a random number generator was used to select one NHS Hospital Trust from each ICS. We also included one Health Board from Scotland and one Health Board from Wales.

### 2.3 Data Collection

Green Plan documents were collected from the NHS Trust websites. We analysed Green Plans from 40 NHS Trusts, representing 40 of the 42 ICS across England. Green Plans were qualitatively examined for any details on plant‐based offerings, strategies to promote these options, and compared to the inclusion of strategies to promote local food and reduce food waste.

Menu information from the hospitals in our sample was requested under the Freedom of Information (FOI) Act 2000. Requests were sent to each hospital for information on the standard menus used in the spring/summer season of 2024, and the catering service provider for each hospital site. A total of 52 FOI requests were submitted, and 40 responses were received. Of these, 34 responses were included in the analysis sample. Six responses were excluded due to insufficient information.

Our scoring system was designed to account for different layouts. If a hospital used a cyclical menu style (i.e., a set menu which is repeated after 1–4 weeks), we only analysed the first week as a representative of the overall sustainability efforts of the hospital. In the case of an À la Carte style, we analysed the whole menu [12]. One researcher collected data from the menus and recounted all entries twice to ensure accuracy.

Additional aspects for which information was collected include the prevalence of processed meat, meat alternatives, and dairy alternatives on hospital menus. These data are not used for the scoring system and are presented at the end of the Results section.

### 2.4 Developing the Scoring System

The scoring system was developed to examine the provision of plant‐based meals, ruminant meat‐based meals, and the use of nudging techniques to encourage the consumption of plant‐forward food choices. A detailed overview of all dimensions included in the scoring system is presented in Table 1. The scoring system was informed by the World Resources Institute's (WRI) ‘The Food Service Playbook for Promoting Sustainable Food Choices’, recommendations from the BDA Nutrition and Hydration Digest, expert consultations, and a review of relevant scientific literature [12, 18].

**Table 1:** Scoring system used to assess hospital menus. Weights are used to calculate a subscore between 0 and 100 for each of the three dimensions ‘main meals’, ‘smaller meals’ and ‘menu presentation’. The final score is then calculated as the subscores' weighted average with weights 3 (main meals), 1 (smaller meals) and 2 (menu presentation). Points are awarded for the provision of vegan meals, vegetarian meals, minimal ruminant meat‐based meals, daily offering of vegan and vegetarian meals and nudging towards lower‐emissions dishes.

Category	Description	Values	Weight	Argument for inclusion and weighting
Food provision (lunch meal)	At least one vegan main provided every day for lunch?	Yes = 100 No = 0	0.5	Offering a vegan lunch main course daily ensures the availability of a lower‐emission meal option each day. Combining the lunch and dinner scores, this dimension has an overall weight of 1.
Food provision (evening meal)	At least one vegan main provided every day for dinner?	Yes = 100 No = 0	0.5	
Food provision (lunch meal)	Percentage of vegan mains for lunch	Between 0 and 100	1	Vegan options have a significant contribution to reducing greenhouse gas emissions (lowest emitting choice). Higher selection rate as a main meal option. Combining the lunch and dinner scores, this dimension has an overall weight of 2.
Food provision (evening meal)	Percentage of vegan mains for dinner	Between 0 and 100	1	
Food provision (lunch meal)	Percentage of vegetarian mains for lunch	Between 0 and 100	0.5	Compared to meat‐based dishes, vegetarian dishes tend to have a lower environmental footprint. Combining the lunch and dinner scores, this dimension has an overall weight of 1.
Food provision (evening meal)	Percentage of vegetarian mains for dinner	Between 0 and 100	0.5	
Food provision (lunch meal)	Percentage of mains for lunch without beef/lamb/goat	Between 0 and 100	0.5	Ruminant meat production has a substantially higher environmental impact compared to other animal‐sourced foods such as chicken and pork. Combining the lunch and dinner scores, this dimension has an overall weight of 1.
Food provision (evening meal)	Percentage of mains for dinner without beef/lamb/goat	Between 0 and 100	0.5	
Food provision (smaller meal)	Percentage of vegan smaller meals	Between 0 and 100	2	Combining the scores for vegetarian and vegan smaller meals, this dimension has an overall weight of 1.
Food provision (smaller meal)	Percentage of vegetarian smaller meals	Between 0 and 100	1	
Menu presentation	Are positive descriptors used on the menu?	Yes = 100 Partially = 50 No = 0	1	Communicating the environmental benefits of choosing plant‐based options can increase the likelihood of them being chosen.
Menu presentation	Are vegan options positioned first on the menu?	Yes = 100 No = 0	1	Listing plant‐rich dishes first on menus can increase the likelihood of that dish being chosen.
Menu presentation	Percentage of vegan or vegetarian dishes that use positive labels.	Regular use ( > 20% of dishes) = 100 Sometimes used (between 0% and 20% of dishes) = 50 No use = 0	0.5	Recommended by the BDA to use language such as ‘plant‐rich’ to describe vegetarian and vegan dishes.
Menu presentation	Percentage of vegan or vegetarian dishes that do not use negative labels	No use = 100 Sometimes used (between 0% and 20% of dishes) = 50 Regularly used ( > 20% of dishes) = 0		Removing unappealing language such as ‘vegetarian’ or ‘vegan’ can increases the likelihood of that dish being chosen. Combining the positive and negative label scores, this dimension has an overall weight of 1. The threshold of 20% for calculating the score was set based on the data.

Each indicator is weighted based on its relative importance. The weighted indicators are then used to calculate three subscores: ‘main meals’, ‘smaller meals’ and ‘menu presentation’. Each subscore is an average of the weighted indicators, with a value between 0 and 100. The final score is calculated as the subscores' weighted average with weights 3 (main meals), 1 (smaller meals) and 2 (menu presentation). For example, meat‐based mains were assigned the lowest scores due to their greater environmental impact compared to fully plant‐based (vegan) meals, while semi‐plant‐based (vegetarian) meals were scored higher than meat‐based mains but lower than vegan means to reflect their relative environmental impact [18, 20]. Overall, higher scores in the resulting subscores ‘main meals’ and ‘smaller meals’ represent a menu that provided more plant‐forward options and can therefore be considered more sustainable.

We assessed the use of behavioural nudging techniques, such as the positioning of plant‐based options on the menu, the use of positive language to describe these dishes, and the absence of terms like ‘vegetarian’ or ‘vegan’ to broaden appeal. Specific techniques were selected based on their applicability to a hospital setting and their evidence base for effectiveness in promoting sustainable choices. We chose nudging techniques from the WRI Playbook that would be applicable to a hospital food environment, as well as from the BDA nutrition and hydration digest, to create four nudging dimensions [12, 18]. These included the following:

1. Communicate the individual environmental impact (e.g., GHG emissions) of a diner switching from a meat to a plant‐rich dish on the menu.
2. List plant‐rich dishes first on the menus to increase the likelihood that they are seen by patients as they read down the list of options.
3. Use positive language such as ‘plant‐based protein’ and ‘plant‐rich’.
4. Avoid using the terms ‘vegetarian’ and ‘vegan’ in the name of dishes so that they appeal to a wider audience.

The resulting nudging subscore ‘menu presentation’ is calculated based on the items in Table 1, each of which evaluates whether the hospital menu adheres to the respective recommendations for incentivising lower‐emission choices. If information on any individual dimension or item is missing, it is excluded from the subscore calculation. The overall subscore is then only calculated provided that at least half of the information from the individual dimensions – determined by the sum of their attributed weights – is available.

Five hospitals did not include smaller meals on their standard menus. In two instances, smaller meals were offered as part of a daily ward‐based selection and, therefore, could not be assessed. In the remaining three cases, no smaller meals were listed on the menu, leaving it unclear whether these hospitals offered smaller meals at all or if they were provided verbally on the ward.

Statistical analysis was limited to descriptive analysis, focusing on the distribution of the overall scores and the three subscores. All analyses were performed with the statistical open‐source software R [21]. Visualisations were created with packages ‘ggplot2’ [22] and ‘gt’ [23].

### 2.5 Ethical Considerations

The study did not require ethical approval as no human participants were involved in the research. The project was formally registered with the University of Winchester [project code UREC240207_Kassam].

### 2.6 Green Plans

The analysis of Green Plans 40 NHS Trusts indicates a limited commitment to increasing plant‐forward food options as part of their Net Zero strategies, with a stronger focus on promoting locally sourced food and reducing food waste. 48% of the plans included at least one aim to increase plant‐based options, and 20% proposed reducing meat consumption through initiatives such as Meat‐Free Mondays or menu alterations. Only 15% outline methods to monitor changes in plant‐based offerings, and 5% include an aim to encourage staff, visitors, or patients to choose plant‐forward options. In comparison, 82% of the Green Plans aim to increase the inclusion of local and seasonal foods on their menus, and 92% address food waste reduction.

### 2.7 Hospital Menus

A total of 36 menus were included in the analysis, received from 34 NHS hospitals trusts. Out of the 36 hospital menus in our sample, 17 were provided by in‐house catering, 14 were contracted out, 3 were a mix of in‐house and contracted out, and 2 did not provide a clear response on their catering provider.

**Figure 1:**
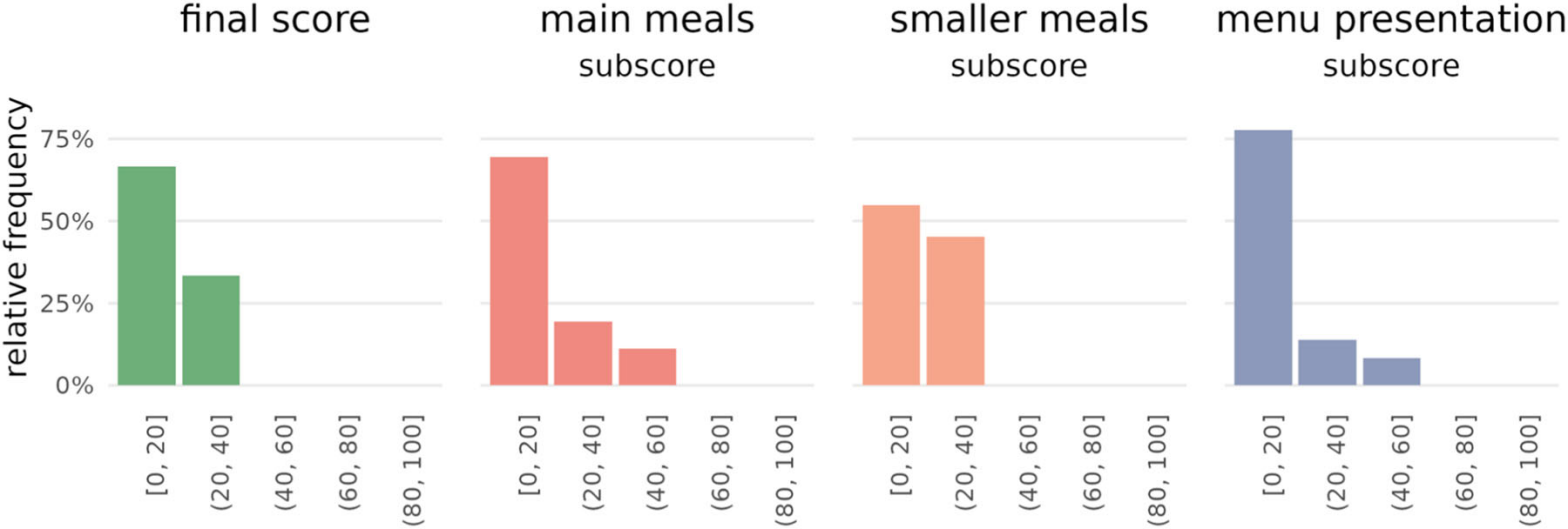
Overall distribution of the final score and the three subscores.

The average hospital score was low with 20 out of 100 points (range 9–38). The overall distribution for the final scores and subscores is presented in Figure 1. Regarding the overall distribution, most hospitals scored between 0 and 20 in their final score and all subscores: main meals, smaller meals and menu presentation.

**Figure 2:**
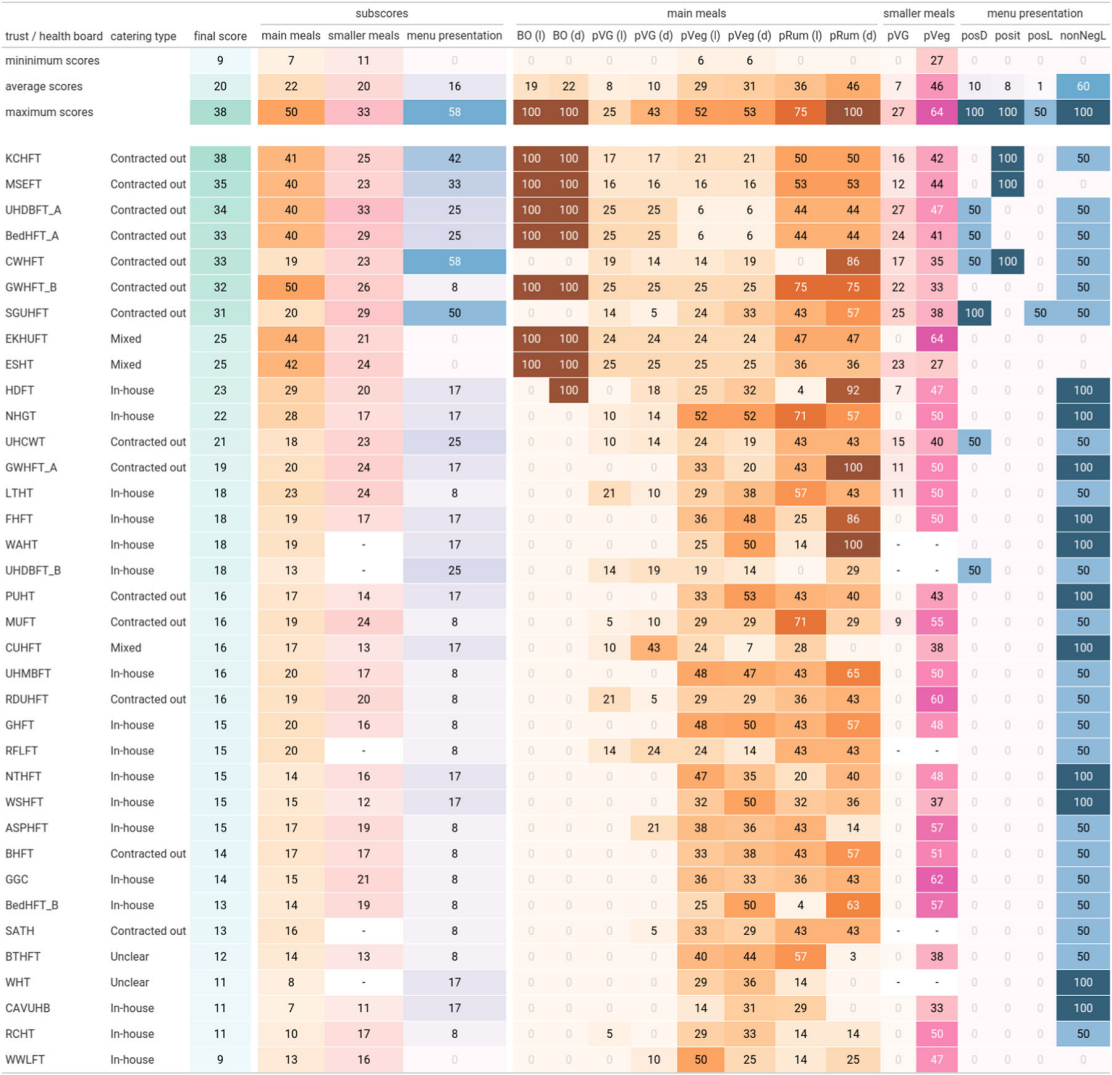
Hospital trusts and their respective hospitals' scores based on the scoring system, ranked from highest score to lowest score. The top three rows show the minimum, average value and maximum of each individual score over all hospitals. The ‘trust/health board’ abbreviations are defined in File S1.

The individual scores and subscores for each hospital are presented in Figure 2. Kings College Hospital London (abbreviated as KCHFT, 38) scored highest, followed by Mid and South Essex (MSEFT, 35), and University Hospitals of Derby and Burton (UHDBFT_A, 34), representing a leading, but overall, only moderate maximum commitment towards increasing and promoting plant‐forward options. The minimum score in the cohort was Wrightington, Wigan and Leigh Teaching Hospitals (WWLFT, 9). The NHS Trust and Health Board abbreviations can be found in File S1.

The average subscore for the main meals was 22. Hospitals scored somewhat better regarding their vegetarian offerings, with average scores of around 30 (range 6–53). 19% (*n* = 7) scored 100 for the lunch base offer, and 22% (*n* = 8) scored 100 for the dinner base offer, representing a fully plant‐based meal provided every day. The highest score achieved for the availability of fully plant‐based lunch and dinner meals was 25 and 43, respectively. 42% (*n* = 15) and 50% (*n* = 18) offered no fully plant‐based mains on their menus for dinner and lunch, respectively.

In contrast to the above results, only 2 hospitals scored 100 for ruminant meals at dinner, based on ruminant‐meat‐based meals having made up a relatively smaller portion of their meal options. Every hospital menu included ruminant meat on their lunch options, with an average score of 36 for lunch meals and 46 for dinner meals. In some cases, hospitals scored 0 for their ruminant‐meat offerings at lunch or dinner, meaning at least a third of their dishes included ruminant meat.

The dimensions with the lowest scores were the provision of fully plant‐based mains for lunch, dinner, and smaller meals, and three of the menu presentation elements: positive and negative labelling and the positioning of plant‐based options, all with an average score ≤ 10. This represents a lack of fully plant‐based meals and of the application of nudging techniques across hospital menus.

### 2.8 Nudging Techniques

The majority of menus in our sample do not incorporate nudging techniques on their menus. The minimum score received for menu presentation was 0, with an average of 16 and a maximum score of 58. Only 8% (*n* = 3) of the hospitals listed the plant‐based options first on the menu. 69% (*n* = 25) hospitals used negative labelling on at least one main dish (lunch or dinner) such as describing the meal as ‘vegetarian’, ‘vegan’ or ‘meat‐free’.

Column name abbreviations: BO (l)/BO (d), base offer: vegan lunch/dinner meal provided daily; pVG (l)/pVG (d), percentage of lunch meals/dinner meals that are vegan; pVeg (l)/pVeg (d): percentage of lunch/dinner meals that are vegetarian; pRum (l)/pRum (d), percentage of lunch/dinner meals without ruminant meat; pVG/pVeg, percentage of smaller meals that are vegan/vegetarian; posD, positive descriptors used on the menu; posit, vegan options positioned first on the menu; posL, percentage of vegan or vegetarian dishes that use positive labels; nonNegL, percentage of vegan or vegetarian dishes that do not use negative labels.

### 2.9 Comparing In‐House and Outsourced Catering Providers

**Figure 3:**
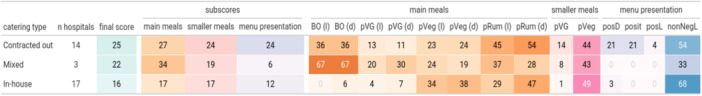
Average final scores, subscores and scores for all the individual dimensions, for in‐house, mixed, and contracted out catering providers (*n* = 34). See Figure 2 caption for the column name abbreviations.

Figure 3 shows the average scores for all contracted out menus, compared to mixed catering and in‐house catered menus. On average, contracted out menus scored higher than in‐house menus, with a score of 25 compared to 16.

### 2.10 Use of Plant‐Based Meat and Dairy Alternatives

Evaluating individual additional dimensions outside the scoring system, 8% (*n* = 3) menus included dairy alternatives (one or more dairy product alternatives excluding milk alternatives) on the standard inpatient menu. We did not assess the provision of non‐dairy milk alternatives as it was not possible to reliably collect this data from the standard inpatient main menus. Meat alternatives were more commonly found on standard inpatient menus, with 72% (*n* = 26) hospitals including at least one dish with a meat alternative across lunch and dinner.

### 2.11 Processed Meat

**Figure 4:**
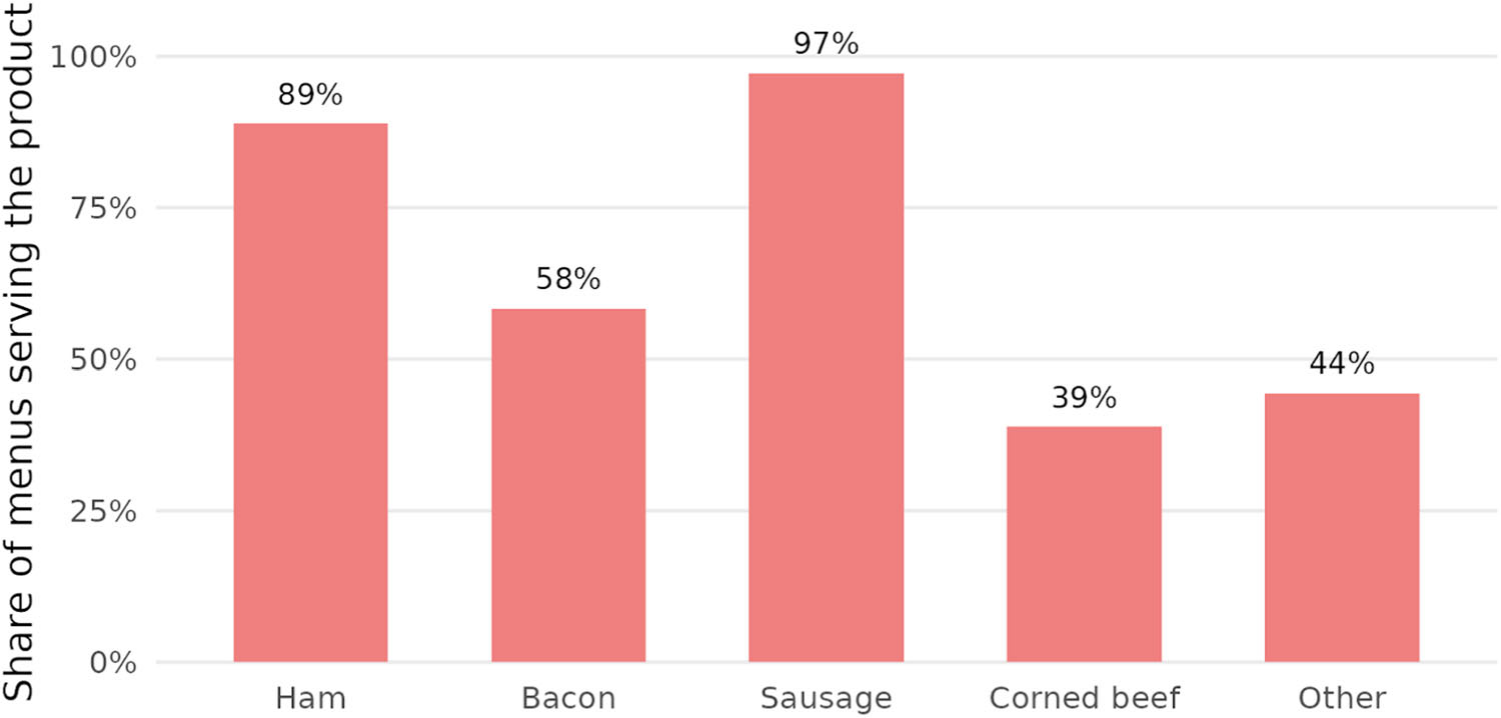
Proportion of menus serving the following processed meats: ham, bacon, sausage, corned beef, and others, on their standard inpatient hospital menu.

Figure 4 presents the proportion of menus regarding their inclusion of each of five types of processed meat. The most commonly used processed meats were sausage (97%), ham (89%) and bacon (58%). Of the 36 menus analysed, all contained at least one meal (lunch or dinner) with processed meat. 58% (*n* = 21) provided at least one dish containing processed meat every day. The average number of items presented as main meals that contained processed meat was 4.6 (range 0–11).

## 3 Discussion

This study introduced a novel sustainability scoring system which evaluates the availability of plant‐ and ruminant meat‐based dishes, and the use of nudging techniques towards plant‐forward dishes on hospital menus in the UK. Overall, hospitals received low scores, mainly due to a limited provision of fully plant‐based dishes and a relatively high availability of ruminant meat. Additionally, all menus contained processed meat, despite being classified as a Group 1 carcinogen [3]. The results suggest a gap between recommendations and current practices in NHS hospitals to include plant‐forward menu items. To the best of our knowledge, this is the first study to examine the provision of plant‐ and animal‐based dishes on NHS hospital menus.

NHS Green Plans present a limited commitment towards transitioning to plant‐forward menus, with few considering implementing methods to monitor changes or nudge staff, visitors, or patients to choose plant‐forward options. Trusts were more likely to commit to the inclusion of local and/or seasonal foods on their menus, or to address food waste reduction as a method to reduce the emissions of their food and catering service. This aligns with evidence that suggests food waste reduction is the most widely explored strategy by hospital food services to improve environmental sustainability [24]. Reducing food waste is important for reducing carbon emissions; one study suggests that food waste might be lower from vegetarian‐ compared to meat‐based meals [25].

Given that NHS hospitals produce approximately 1,543 kt CO2e annually from food and catering services—representing 6% of their total emissions—this is a key area for climate action. Plant‐based meals have a significantly lower environmental impact than meat‐based meals [20, 26], and their implementation in hospital menus can reduce carbon emissions [20, 25, 27, 28]. Hospital menus in the Netherlands showed that vegetarian meals had on average, 50% lower GHGE compared to animal‐based meals and beef contributed significantly to GHGE [29]. The adoption of a plant‐based by default programme for inpatient menus at NYC Health + Hospitals led to an estimated 36% reduction in carbon emissions from all meals [27]. In Turkey, menus suitable for the Mediterranean diet could achieve a 2.2%–23.4% reduction in the carbon footprint and 37.5%–58.6% reduction in the water footprint [28].

Several factors may explain the low scores observed across the menus in this study, including food culture, patient preferences, and operational constraints in menu design, which may prevent hospitals from adapting their menus in response to new guidance. The NHS is also constrained by financial and workload pressures, and an independent review of NHS hospital food found that NHS trusts do not always see food as a priority, leading to insufficient investment and budget cuts in catering services [11]. Hospital food service stakeholders report several barriers to increasing the proportion of plant‐to‐animal protein in hospital patient menus, such as concerns about patient acceptance, potential impact on protein intake and malnutrition rates, and the cost of plant‐based protein foods [30]. However, most supported menu changes and they also recognised advantages, including improvements in the menu's nutritional profile, health benefits, lower cost for legumes compared to meat, reduced greenhouse gas emissions and the opportunity to use the menu as an education tool [30]. Registered dietitians (RDs) may share similar perceptions, as indicated by a recent survey on attitudes toward whole‐food, plant‐based diets (WFPBDs). RDs had concerns over the risk of malnutrition and micronutrient deficiencies, with 75% incorrectly believing that plant proteins are incomplete. Only 33% felt supported when advocating for a WFPBD in their workplace, yet the majority agreed that WFPBDs should be included as a recognised therapeutic dietary option in hospitals for appropriate patients [31]. Similar barriers, including patient preferences and difficulties with staff and planning menus, have also been reported by food service directors regarding the integration of more plant‐based meals in US veteran hospitals [32].

Despite the perceived barriers, patients' acceptance of plant‐based meals could be higher than anticipated. A survey of patients at King's College Hospital, London, found that the majority did not consider serving processed red meat (74%) or unprocessed red meat (66.1%) in hospitals important to them. Most patients (77.9%) were neutral or supportive of removing processed red meat from menus to reduce the risk of cancer, and 67% were supportive of removing unprocessed red meat from menus to combat climate change [33]. The plant‐based by default programme at NYC Health + Hospitals also demonstrated strong acceptance and satisfaction among patients for plant‐based meals; 95% of eligible patients chose plant‐based options and did not request alternate meals, and 90% of patients who received the plant‐based meals reported being satisfied with their choice [27]. In other settings in the United States, more plant‐based meals have been successfully incorporated within in‐patient settings [34]. Expanding plant‐based meals would still allow patients the freedom to choose between meat‐based and plant‐based meals according to their preferences.

In our study, menus contracted out to private catering companies scored, on average, better than in‐house menus and several factors may explain this result. Private catering companies often set their own climate targets, with some committing to reducing catering emissions and reaching net zero by 2030 [35], ahead of the NHS' target to reach net zero by 2045 [36]. For example, Compass Group UK & Ireland has set the goal of a 40% switch towards plant‐based proteins by 2030 [37], a target more detailed than any identified in the NHS Green Plans in this sample. Additionally, the UK government has introduced requirements for catering companies to commit to net‐zero emissions and produce carbon reduction plans to secure catering contracts [38]. Private catering companies may also have more resources to develop and update menus with plant‐forward options [11].

The main requirement of hospital menus is to meet the nutritional requirements of patients, including those with complex therapeutic dietary needs. When incorporating sustainable meals, it is important to ensure they meet the nutritional needs of patients. Protein intake is often considered a nutrient of concern for plant‐based meals, however, plant‐based diets are fully adequate to meet protein requirements, given they are planned appropriately [39]. Evidence from hospitals in the Netherlands suggests that vegetarian meals may fail to meet protein requirements without adequate planning or ingredient selection however, adding or modifying ingredients improves the protein quality [29]. Properly designed plant‐based meals are not only capable of meeting protein requirements but are also associated with long‐term health benefits, such as lower risk of cardiovascular disease and type 2 diabetes [40, 41, 42]. Few studies have examined the nutrient differences between plant‐based and animal‐based meals in hospitals. Recent estimates suggest that in UK hospitals, 44% of patients were at risk of malnutrition, and there is emerging evidence from a community setting that replacing animal‐based protein with plant protein may improve malnutrition [43]. Plant‐based meals are suitable for a wide number of therapeutic orders, including a consistent carbohydrate diet, cardiac diet, low sodium diet, low‐fat diet, high protein/calorie diet, and are inclusive to all religious, cultural and dietary preferences.

Despite recommendations from the BDA Nutrition and Hydration Digest to implement nudging techniques to encourage the consumption of plant‐based dishes, we found limited implementation in the hospitals within our sample. Research in other public sector settings suggests that positively framed language and the strategic placement of plant‐based options can increase their selection [18]. In a hypothetical hospital setting, adjustments to menu design modestly influenced food choices and satisfaction among meat‐eaters. The study found that increasing the availability of vegetarian options and including health recommendations could increase the selection of vegetarian dishes [44]. Nudging provides a promising mechanism to influence patient behaviour without requiring significant effort or deliberate decision‐making on their part. By reshaping the choice environment – making plant‐based options more accessible, appealing, and the default choice – it is possible to promote healthier and more sustainable food choices within the hospital setting. Given the limitations in resources, this could be an underutilised tool to promote a more environmentally sustainable hospital food environment. However, to the best of our knowledge, there is no research directly assessing the effectiveness of such nudging techniques on inpatient populations, which limits the generalisability of these findings.

This study provides one of the few assessments of hospital food environments in the UK with a focus on plant‐based food options and the use of behavioural nudging techniques. Our sample of 36 hospitals represents most regions in England, which incorporates both rural and urban areas of differing socioeconomic status. By using a novel scoring system developed from existing evidence‐based frameworks, the study provides a structured and systematic evaluation of hospital menus across multiple NHS Trusts. Given the urgency of shifting away from meat‐heavy to plant‐forward diets to meet climate targets, this framework offers a tool to track progress and understand how hospitals are supporting more sustainable food choices. Finally, this study highlights actionable areas for improvement, offering practical insights that could inform future policy changes within NHS food services, supporting both health and environmental goals.

### 3.1 Limitations

This study has several limitations that should be noted. Firstly, the scoring system used was developed specifically for this study and has not been externally validated. While it was informed by evidence‐based frameworks and expert consultation, future research could refine and validate this tool across broader healthcare settings. The included menus may not be representative of what each hospital offers year‐round, as menu rotations and seasonal variations were not accounted for. Certain behavioural nudging techniques that are known to be relevant to hospital settings were not included in this study, such as reducing the amount of meat in popular recipes by incorporating more plant protein, as it was not possible to identify such dishes from the menus provided. In addition, it was not possible to correlate these nudge techniques with meal uptake data, as requests for this information were inadequately fulfilled.

### 3.2 Recommendations and Future Research

It is now critical that hospitals balance the nutritional needs of patients with low carbon requirements. Our analysis reveals there is a gap in meeting national and international sustainability guidelines [6, 12]. To achieve the NHS's goal of net zero emissions by 2045, NHS hospitals should integrate more sustainable options into hospital food services. Based on the low (sub)scores obtained across all menus, several strategies for improving menus can be identified. Reducing the frequency of red meat options, particularly ruminant meats like beef and lamb, is an urgent priority. Additionally, hospitals should look to replace animal‐based proteins with plant‐based protein in familiar recipes, remove processed meat entirely, move plant‐based options above animal‐based options, add more plant‐forward options, and remove negative language from plant‐based dishes. NHS hospitals that work with outsourced catering providers, who play a key role in menu design, have the potential to influence the implementation of more effective nudging strategies. Future research should focus on including smaller and specialist hospitals in the UK, to more accurately depict the state of the food environment across the UK. Ongoing monitoring and evaluation of NHS hospital menus is essential to ensure progress towards sustainability targets. As Trusts work to improve their menus, the scoring system can be re‐applied to assess how effectively hospitals are encouraging more sustainable meal choices.

## 4 Conclusion

Despite an appetite for change for plant‐forward menus in healthcare, this study reveals current hospital food is far from what's desired to support aims of a net‐zero healthcare system. Beyond the UK healthcare system, there is a global need to shift dietary patterns towards plant‐forward diets, hospitals provide teaching moments to facilitate this change and should do more to utilise this position.

## Author Contributions

Isabelle Sadler, Alexander Bauer and Shireen Kassam contributed to the study conception, study design, data analysis, interpretation of the findings and the writing of the manuscript.

## Conflicts of Interest

Isabelle Sadler is a member of Plant Based Health Professionals UK. Shireen Kassam is the founder and director of Plant Based Health Professionals UK. Alexander Bauer declares none.

### Peer Review

The peer review history for this article is available at https://www.webofscience.com/api/gateway/wos/peer-review/10.1111/jhn.70019.

## Supporting information

Supporting information.

## Data Availability

The data that support the findings of this study are available from the corresponding author upon reasonable request.
