# Supplementary material for: How Sustainable Are Hospital Menus in the United Kingdom? Identifying Untapped Potential Based on a Novel Scoring System for Plant‐Based Provisions
Source: J Hum Nutr Diet. 2025 Feb 3;38(1):e70019. doi: 10.1111/jhn.70019 (PMC11789209; doi:10.1111/jhn.70019)
Supplement: Supplementary file 1 — Supporting information. [file JHN-38-0-s001.docx]

Supplementary Material

Supplementary File 1

Table S1. A list of NHS Hospital Trust abbreviations and their official names.

| SGUHFT | St George's University Hospitals |
| --- | --- |
| GGC | Greater Glasgow & Clyde |
| CAVUHB | Cardiff & Vale UHB |
| RFLFT | Royal Free London |
| MUFT | Manchester University |
| UHCWT | University Hospitals Coventry & Warwickshire |
| KCHFT | Kings College Hospital |
| PUHT | Portsmouth Hospitals University |
| LTHT | The Leeds Teaching Hospitals |
| BTHFT | Bradford Teaching Hospitals |
| NHGT | Northampton General Hospital |
| BHFT | Barnsley Hospital |
| WHT | Walsall Healthcare |
| UHMBFT | University Hospitals of Morecambe Bay |
| UHDBFT_A | University Hospitals of Derby and Burton |
| UHDBFT_B | University Hospitals of Derby and Burton |
| NTHFT | The Newcastle Upon Tyne Hospitals |
| BedHFT_A | Bedfordshire Hospitals |
| BedHFT_B | Bedfordshire Hospitals |
| ASPHFT | Ashford and St Peters |
| MSEFT | Mid and South Essex NHS Foundation Trust |
| WAHT | Worcestershire Acute Hospital NHS Trust |
| WWLFT | Wrightington, Wigan and Leigh Teaching Hospitals NHS Foundation Trust |
| SATH | Shrewsbury and Telford Hospital NHS Trust |
| WSHFT | West Suffolk NHS Foundation Trust |
| HDFT | Harrogate and District NHS Foundation Trust |
| ESHT | East Sussex Healthcare NHS Trust |
| GWHFT_A | Great Western Hospitals |
| GWHFT_B | Great Western Hospitals |
| CWHFT | Chelsea & Westminster Hospital NHS FT |
| RDUHFT | Royal Devon University Healthcare |
| RCHT | Royal Cornwall Hospitals NHS Trust |
| EKHUFT | East Kent Hospitals University NHS Foundation Trust |
| GHFT | Gloucestershire Hospitals NHS Foundation Trust |
| FHFT | Frimley Health NHS Foundation Trust |
| CUHFT | Cambridge University Hospitals NHS Foundation Trust |
